# Effect of transcranial direct current stimulation on cognitive function in stroke patients

**DOI:** 10.1186/s41983-018-0037-8

**Published:** 2018-11-06

**Authors:** Hussien Ahmed Shaker, Salah Abd Elmonem Sawan, Ebtesam Mohamed Fahmy, Rania Shehata Ismail, Shymaa Abd Elhamid Abd Elrahman

**Affiliations:** 1 Department of Physical Therapy for Neuromuscular Disorder and its Surgery, Faculty of Physical Therapy, Cairo University, Giza, Egypt; 2 Neurology Department, Faculty of Medicine, Cairo University, Giza, Egypt

**Keywords:** Stroke, Transcranial direct current stimulation, Cognitive functions

## Abstract

**Background:**

Cognitive impairment after stroke is common and can cause disability with major impacts on quality of life and independence. Transcranial direct current stimulation may represent a promising tool for reconstitution of cognitive functions in stroke patients.

**Objectives:**

This study aimed to investigate the effect of transcranial direct current stimulation on cognitive functions in stroke patients.

**Patients and methods:**

Forty male stroke patients were included. Patients were divided randomly into two equal groups (A and B). Group A received transcranial direct current stimulation (tDCS) in combination with selected cognitive training program by RehaCom. Group B received sham transcranial direct current stimulation in combination with the same cognitive training program.

Cognitive evaluation and functional independence measure (FIM) were done for all patients before and after treatment.

**Results:**

There was a significant improvement in the scores of attention and concentration, figural memory, logical reasoning, reaction behavior, and FIM post treatment in both groups; the improvement was significantly higher in group A compared to group B.

**Conclusion:**

tDCS is a safe and effective neuro-rehabilitation modality that improves post stroke cognitive dysfunctions. Moreover, tDCS has a positive impact on performance of daily activities.

## Introduction

After a stroke, as many as 55% of people have deficits in episodic memory, up to 40% show deficits in executive functions, and 23% of people have language deficits [1]. These cognitive deficits have a significant impact on the ability to carry out activities of daily life and may interfere with successful social and occupational reintegration [2]. The elevated prevalence of cognitive dysfunction associated with stroke makes cognitive rehabilitation essential [3].

RehaCom is a computerized presentation and guidance that create a tailored cognitive training for patients. RehaCom training improves cognitive abilities of patients to make it possible to meet demand for care, improves quality of life, and optimizes performance in impaired functions [4, 5].

Transcranial direct current stimulation (tDCS) is an emerging technique of noninvasive brain stimulation that has been proposed as a tool to improve cognitive function after ischemic stroke [6, 7]. Park and colleagues [8] found that the concomitant use of anodal tDCS with a computer-assisted cognitive rehabilitation program had a significant effect on improving attention in post stroke patients with mild to moderate cognitive dysfunction.

The primary mechanism of tDCS is depolarizing or hyperpolarizing neural tissue, inducing significant changes in the resting membrane potential [9, 10]. tDCS may provide a useful tool to modulate synaptic plasticity in stroke [11].

Neuroimaging have demonstrated that the right and left dorsolateral prefrontal cortex (DLPFC) are involved in cognitive function. So, stimulation of this area can improve cognition in patients with cognitive impairment [12].

This study was designed to investigate the efficacy of transcranial direct current stimulation on improving cognitive impairment in stroke patients.

## Subjects and methods

### Subjects

This randomized controlled single-blinded trial was conducted on 40 male patients with first-ever ischemic cerebrovascular stroke in the period from January to November 2017. Patients were selected from the outpatient clinic of Faculty of Physical Therapy, Cairo University, and neurology outpatient clinic of Cairo University Hospitals. The aim and procedures of the study were explained to every participant, and an informed consent was obtained before being enrolled in the study. The study was approved by the ethical committee of Department of Physical Therapy for Neuromuscular Disorders and its Surgery, Faculty of Physical Therapy, Cairo University, registration number NO:P.T.REC/012/001071.

Patients were randomly divided into two equal groups (A, B). Group A was treated with transcranial direct current stimulation (tDCS) in combination with selected cognitive training program. Group B was treated with sham transcranial direct current stimulation in combination with the same cognitive training program.

### Included in this study

Included in this study were patients with ischemic cerebrovascular stroke in the domain of carotid system diagnosed clinically and confirmed by CT and/or MRI of the brain, their age ranged from 40 to 60 years old to minimize the effect of age on cognition, duration of illness at least 6 months from stroke onset, educated patients at least 10 years of formal education, degree of spasticity was 1 and 1+ according to Modified Ashworth Scale, grade of muscle power was 3 according to manual muscle test, mini mental state examination score ranged from 19 to 24 (mild cognitive impairment), functional independence measure score ranged from 84 to 99, Beck depression inventory score from 0–9, and normal hearing and vision.

### Excluded from this study

Excluded from this study were patients with recurrent cerebrovascular stroke or concomitant neurological disorders that may affect cognition, psychiatric disorders, drug or alcohol abuse, and seizure disorders; patients receiving drugs that may affect cognitive functions (anti-depressants, anti-epileptics, sedatives, muscle relaxants); and patients with medical illness that may affect cognitive functions (thyroid, renal or hepatic disorder) and a presence of wounds in the skin of the skull.

## Methods

For assessment:

1. RehaCom system: Assessment of cognition by RehaCom was carried out at the cognition laboratory of Faculty of Physical Therapy, Cairo University. RehaCom model is Schuhfried, model No. 454, D- 14482 posted am, Karl-Liepknecht, Austria. RehaCom is a software package that is used for the assessment of cognitive functions. RehaCom is operated with a panel. The RehaCom panel is a special keyboard with simple, large, and clear keys. The input panel has six big keys, four large white keys expressing the up, down, right, and left direction to choose the right answer and two large green keys (OK) to confirm choice. There are also two special keys, red key for emergent stop and yellow one for more information about the procedure. Four cognitive domains were assessed: attention and concentration, figural memory, reaction behavior, and logical reasoning [13].
2. Functional independence measure (FIM): which are a widely used measure of disability and the impact of disease or impairment on performance of daily activities. It was designed to assess the degree of assistance required by a person with a disability to perform daily activities (the burden of care). It has been reported to be reliable and valid. The scale scores range from 18 to 126, the higher the score, the better the independence [14].

For treatment:

1. Transcranial direct current stimulation device: The tDCS device model is Apex Electronics, LLC. 1737 Union St.#171. Schenectady, NY 12309. USA 1-877-915-TDCS. The device consists of power supply (nine volts of direct current), a pair of metal conductive electrodes soaked in sponge pocket, cables used to connect the electrodes to the tDCS device, saline solution, and head bands to stabilize the electrodes to the head [15].
2. RehaCom for cognitive training: RehaCom is a computerized program instituted in the International Neurological Restoration Center for rehabilitation of brain injury. RehaCom is a software package that enables to train different cognitive areas. Each program consisted of several levels of difficulty with a sufficient item pool to make sure that a patient works only on tasks corresponding to his current abilities.

Treatment procedures:

1. Transcranial direct current stimulation

The skin of the scalp was inspected for any irritation, cuts, or lesions to avoid tDCS stimulation over damaged skin. To increase conductance, the hair was removed away from the site of stimulation and the surface of the skin was cleaned to remove any lotion or dirt. The electrodes were placed according to the International 10–20 system for EEG electrode placement. The anode electrode was placed over F3 and F4 to stimulate the right and left dorsolateral prefrontal cortex (DLPFC). The cathode was placed over the contralateral supraorbital area. The electrodes were covered with saline-soaked sponges to decrease impedance [16].

2) Cognitive training by RehaCom

The patient was trained on using the device to be familiar with it before starting the session. During training, the patient received information about the quality of performance by means of simple depictions on the monitor. This information was an item-related feedback which can be positive or negative.

*Attention and concentration training*: the training was based on the pattern and comparison method. The patient had to find out which picture from a matrix corresponds exactly to the sample object. A total of 29 picture pools, each of which contains 16 pictures, had been set up. Different types of objects were represented on these pictures. Either concrete objects (fruits, animals, faces, etc.), geometric objects (circles, rectangles, triangles in different sizes and order), or letters and numbers were displayed.

*Figural memory training*: pictures of concrete objects were displayed. The patient should memorize the pictures of all these objects and terminates the “learning phase” by pressing the OK button. Then, the pictures roll by on the screen from left to right as if on convey or belt. Whenever a picture for an object of the “learning phase” rolls into the box at the edge of the screen, the patient should press the OK button.

*Logical reasoning training*: the patient should add the correct symbol to a row of symbols that had been assembled according to a specific rule*.* From various symbols (“response pool”), patients should select the one which was correctly continuing a given sequence. A sequence of symbols (circles, triangles, squares, etc.) of different shape, color, and size assembled according to a rule was displayed on the screen. When the patient responds incorrectly, specific feedbacks with respect to the type of error were displayed.

*Reaction behavior training*: this program trained the reaction behavior to signals. On the edge of the screen, there were pictures of traffic signs. Next to it, a key of the RehaCom panel was displayed which should be pressed by the patient when the respective traffic sign appears in the middle of the monitor. In the learning phase, the patient was presented with the pictures of the target stimuli (traffic signs) and the corresponding reaction keys. By pressing the OK button, the patient could independently terminate the learning phase and proceed to the training. In higher levels of difficulty, also irrelevant traffic signs were displayed.

*Treatment procedures*: in group A, patients received treatment for 1 month, three sessions per week (every other day). The duration of each session was 60 min, divided into 30 min of tDCS (2 mA) application followed by 30 min of RehaCom training. In group B, patients received sham tDCS and the same cognitive training program as group A. In sham tDCS, real stimulation was applied for 5 s; then, stimulation was turned off.

### Statistical analysis

The collected data were coded, tabulated, and statistically analyzed using IBM SPSS statistics (Statistical Package for Social Sciences) software version 22.0, IBM Corp., Chicago, USA, 2013. Comparison between scores before and after treatment within the same group was performed using paired *t* test. Comparison between mean values of different variables in both groups (A, B) was performed using unpaired *t* test. *p* value < 0.05 was considered significant, and *p* value < 0.01 was considered highly significant.

## Results

### General characteristics

The mean age was 54.45 ± 4.68 years for group A and 53.05 ± 6.32 years for group B. The mean duration of illness was 14.05 ± 1.53 months for group A and 16.55 ± 2.78 months for group B. There was no significant difference between both groups in mean age and duration of illness (*p* value = 0.436, 0.085 respectively).

Group A included 14 school graduates and 6 university graduates (70% and 30% respectively). Group B included 16 school graduates and 4 university graduates (80% and 20% respectively). There was no significant difference between both groups regarding level of education (*p* value = 0.465).

### Scores of attention concentration level (ACL) within each group

There was a significant increase in the mean ACL scores after treatment in groups A and B (*p* = 0.000). The percentage of improvement was 97.59% in group A and 36.47% in group B (Table 1).

**Table 1:** Comparison of mean scores of attention and concentration level pre and post treatment within each group

Parameter	Score of attention and concentration level			
	Group A		Group B	
	Before	After	Before	After
Mean ± SD	4.15 ± 0.99	8.2 ± 1.36	4.25 ± 0.79	5.8 ± 0.83
Improvement %	97.59%		36.47%	
*t* value	− 15.8		− 9.13	
*p* value	0.000**		0.000**	

**Highly significant

### Scores of figural memory level (FML) within each group

There was a significant increase in the mean FML scores after treatment in groups A and B (*p* = 0.000). The percentage of improvement was 78.08% in group A and 27.63% in group B (Table 2).

**Table 2:** Comparison of mean scores of figural memory level pre and post treatment within each group

Parameter	Score of figural memory level			
	Group A		Group B	
	Before	After	Before	After
Mean ± SD	3.65 ± 0.81	6.5 ± 1.19	3.8 ± 0.62	4.85 ± 0.81
Improvement %	78.08%		27.63%	
*t* value	− 12.26		− 7.76	
*p* value	0.000**		0.000**	

**Highly significant

### Scores of logical reasoning level (LRL) within each group

**Fig. 1:**
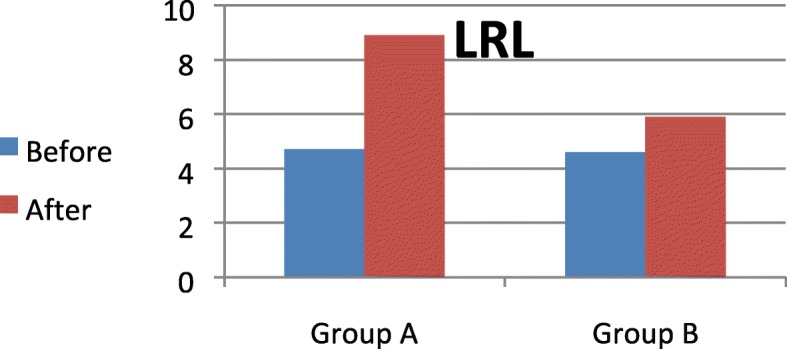
Scores of LRL pre and post treatment within each group. LRL, logical reasoning level

There was a significant increase in the mean LRL scores after treatment in groups A and B (*p* = 0.000). The percentage of improvement was 89.36% in group A and 28.26% in group B (Fig. 1).

### Scores of reaction behavior level (RBL) within each group

There was a highly significant increase in the mean RBL scores after treatment in groups A and B (*p* = 0.000). The percentage of improvement was 99.05% in group A and 35.29% in group B (Table 3).

**Table 3:** Comparison of mean scores of reaction behavior level pre and post treatment within each group

Parameter	Score of reaction behavior level			
	Group A		Group B	
	Before	After	Before	After
Mean ± SD	5.3 ± 0.73	10.55 ± 1.93	5.1 ± 0.79	6.9 ± 1.12
Improvement %	99.05%		35.29%	
*t* value	− 12.25		− 7.62	
*p* value	0.000**		0.000**	

**Highly significant

### Comparison of scores of ACL, FML, LRL, and RBL after treatment between both groups

**Fig. 2:**
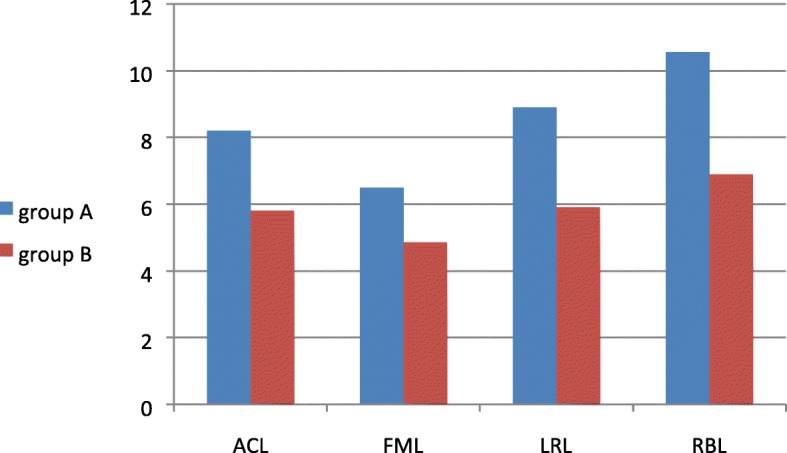
Comparison of the mean scores of ACL, FML, LRL, and RBL after treatment between groups. ACL, attention and concentration training; FML, figural memory training; LRL, logical reasoning level; RBL, reaction behavior training

There was a highly significant difference in the mean scores of ACL, FML, LRL, and RBL after treatment between groups A and B (*p* = 0.000), being significantly higher in group A (Fig. 2).

### Comparison of mean scores of FIM within each group

There was a highly significant increase in the mean FIM score after treatment in groups A and B (*p* = 0.000). The percentage of improvement was 20.99% in group A and 8.32% in group B (Table 4).

**Table 4:** Comparison of the mean score of FIM before and after treatment within each group

Parameter	Score of functional independent measure			
	Group A		Group B	
	Before	After	Before	After
Mean ± SD	95.3 ± 4.78	115.3 ± 9.71	95.6 ± 4.26	103.55 ± 5.48
Improvement %	20.99%		8.32%	
*t* value	− 14.65		− 7.92	
*p* value	0.000**		0.000**	

**Highly significant

### Comparison of mean scores of FIM after treatment between groups

There was a highly significant increase in the mean score of FIM in group A compared to group B after treatment (*p* value = 0.000) (Table 5).

**Table 5:** Comparison of the mean score of FIM before and after treatment between groups

	Score of functional independent measure			
	Group A	Group B	*t* value	*p* value
Before	95.3 ± 4.78	95.6 ± 4.26	− 0.209	0.836
After	115.3 ± 9.71	103.55 ± 5.48	4.71	0.000**

**Highly significant

## Discussion

There is increasing prevalence of cognitive deficits following stroke. These deficits may interfere with social and occupational activities of stroke patients which make cognitive rehabilitation essential [2, 3].

This study aimed to investigate the efficacy of transcranial direct current stimulation in improving cognitive impairment in stroke patients. Patients were divided into two equal groups (A and B). Group A received transcranial direct current stimulation (tDCS) in combination with selected cognitive training program by RehaCom. Group B received sham transcranial direct current stimulation in combination with the same cognitive training program.

The present study revealed significant improvement in attention and concentration in both groups after treatment, especially group A, which agreed with Shin and colleagues [17] who stated that brain stimulation via tDCS increased attention. Also, Pope [18] demonstrated that tDCS enhances cognitive processes by exciting brain regions involved in attention. On the contrary, Roy and colleagues [19] reported that attention deficits are a common consequence of brain damage and the application of 1.5 mA anodal tDCS on right parietal cortex has been shown to modulate spatial attention while tDCS on DLPFC did not modulate attention.

In the current study, there was a significant improvement in mean scores of figural memory in both groups. The improvement was significantly higher in group A who received tDCS on DLPFC. This was consistent with the opinion of Jahanshahi and colleagues [20] who stated that stimulation of DLPFC improves cognition functions such as working memory. Nitsche and colleagues [21] also reported that tDCS enhances cortical excitability and facilitates the learning process and DLPFC has been proposed in learning, information storage, and retrieval. Moreover, Fregni and colleagues [22] stated that tDCS manipulates performance in a variety of cognitive domains and should be considered as a neuro-rehabilitation tool for patients with post stroke cognitive deficits and that anodal stimulation increases the excitability of prefrontal cortex which improve performance of working memory. Also, Felipe and colleagues [23] reported that the left DLPFC anodal stimulation leads to enhancement of working memory performance and improvement of cognitive functions; this effect depends on the stimulation polarity and is specific to the site of stimulation. Ohn and colleagues [24] concluded that anodal tDCS administered to the left DLPFC has a positive effect on working memory. Moreover, Javadi and colleagues [25] applied tDCS over the dorsolateral prefrontal cortex during a memory performance task and reported that anodal tDCS significantly increased behavioral performance when subjects had to memorize words. Elsner and colleagues [26] also reported that tDCS improves working memory and cognitive abilities in people with stroke by modulating excitability of the corresponding brain areas.

The current study revealed significant improvement in mean scores of logical reasoning in both groups; the improvement was significantly higher in group A compared to group B. This finding agreed with Dockery and colleagues [27] who stated that tDCS improves planning ability. Also, Sarkis and colleagues [28] concluded that anodal tDCS over dorsolateral prefrontal cortex improved cognitive processes including planning, problem solving, and reasoning. Moreover, Hartwigsen and colleagues [29] reported that 2 mA of anodal tDCS over left DLPFC as compared with sham tDCS leads to improved performance in a decision task and facilitates behavioral response.

In the present study, there was a significant improvement in mean scores of reaction behavior in both groups especially in group A. These results came in agreement with the opinion of Antal and colleagues [30] who reported that tDCS enhances cognitive functions. Hummel and colleagues [31] also reported that cognitive functions might be modulated more effectively by tDCS. Sparing and colleagues [32] stated that transcranial direct current stimulation tDCS with constant current of 2 mA can increase cortical excitability by polarization of the underlying brain tissue and enhance cognitive function.

The current study revealed significant improvement in mean scores of functional independent measure (FIM) in both groups. The improvement was significantly higher in group A compared to group B. This agreed with Antal and colleagues [30] who reported that application tDCS in the rehabilitation of brain injuries improves performance. The authors suggested that tDCS modulates excitability of the cortex in human subjects, and anodal stimulation increases spontaneous firing rates of cortical cells during and after stimulation. Moreover, due to the large size of stimulating electrode, other brain areas may be activated.

Fregni and colleagues [22] stated that tDCS increased activity in the affected hemisphere in stroke patients which can promote functional recovery. Moreover, Hummel and colleagues [31] reported that tDCS has a beneficial effect on hand function in the paretic hand of chronic stroke patients. Elsner and colleagues [33] suggested that tDCS modulates cortical excitability and might improve activity of daily living and function after stroke. Sarkis and colleagues [28] also reported that cognitive enhancement improves function and quality of life.

Sparing and colleagues [32] reported that tDCS has relatively low spatial focality due to large tDCS electrode sizes. Meinzer and colleagues [34] also stated that tDCS induced changes in brain activity in large-scale networks. Accordingly, it was suggested that anodal tDCS with low frequency is not restricted to the targeted area but also spreads to functionally connected brain areas.

In this study, tDCS was applied on F3 and F4 to stimulate the right and left DLPFC. This agreed with Mottaghy and colleagues [35] who demonstrated that the right and left DLPFC are involved in working memory. Berryhill and colleagues [36] concluded that anodal tDCS applied on F3 or F4 improves performance of working memory in highly educated subjects. Sarkis and colleagues [28] stated that F3 and F4 are the most common electrode placement positions in the studies attempting to enhance cognition using tDCS. Baschi and colleagues [37] concluded that anodal tDCS applied over F3 with intensity 2 mA activated DLPFC. Mir-Moghtadaei and colleagues [38] also reported that DLPFC is represented by F3-F4 in respect to the international 10-20 electroencephalography (EEG) electrodes.

The effects of tDCS on brain tissue were probably induced by increased accumulation of calcium ions and modification of membrane polarization leading to increased cortical excitability after the end of stimulation. Studies of cellular excitability indicate prolonged excitatory potentials and enhanced ability to form long-term excitable responses to inputs. This is termed long-term potentiation (LTP). Stimulation using transcranial direct current enhances LTP and improves stroke recovery [12, 39].

## Conclusion

In view of the results of this study, it could be concluded that tDCS is a safe and effective neuro-rehabilitation modality that improves cognitive functions in several domains including attention and concentration, figural memory, logical reasoning, and reaction behavior. Moreover, tDCS has a positive impact on performance of daily activities. So, it is recommended to use it in the rehabilitation of post stroke cognitive dysfunctions.

## Abbreviations

ACL: Attention concentration level
DLPFC: Dorsolateral prefrontal cortex
EEG: Electroencephalography
FIM: Functional independence measure
FML: Figural memory level
LRL: Logical reasoning level
LTP: Long-term potentiation
RBL: Reaction behavior level
tDCS: Transcranial direct current stimulation
